# Incidence and risk factors of hip dislocation in children with cerebral palsy: A systematic review and pooled analysis

**DOI:** 10.1016/j.jcot.2025.103141

**Published:** 2025-07-28

**Authors:** Gabriele Giuca, Ilaria Sanzarello, Daniela Alessia Marletta, Salvatore Calaciura, Matteo Nanni, Danilo Leonetti

**Affiliations:** Section of Orthopaedics and Traumatology, Department of Biomedical Sciences and Morphological and Functional Images, University of Messina, 98122 Messina (ME) Italy

**Keywords:** Cerebral palsy, Hip dislocation, Incidence, Risk factors, GMFCS

## Abstract

**Background:**

Hip displacement and dislocation are among the most disabling musculoskeletal sequelae of cerebral palsy (CP) yet reported incidence and risk-factor estimates vary widely across studies. We undertook a systematic review and pooled analysis to quantify cumulative incidence across Gross Motor Function Classification System (GMFCS) strata and identify reproducible clinical and radiographic predictors.

**Methods:**

A protocol was registered in PROSPERO (CRD420251026860). MEDLINE (PubMed), Embase, CINAHL and CENTRAL were searched from inception to March 30, 2025. Eligible longitudinal studies enrolled ≥30 children with CP aged 2–18 years, provided ≥2 years’ follow-up without confounding hip-directed intervention, and reported migration percentage (MP) data or equivalent permitting derivation. Hip displacement and dislocation were harmonized as MP >30 % and >50 %, respectively. Study quality was appraised with ROBINS I. Proportions were stabilized with the Freeman–Tukey double-arcsine transformation and pooled in random-effects (REML) models; odds ratios (ORs) for candidate predictors were combined using inverse-variance random-effects methods. Heterogeneity (I^2^, τ^2^), prediction intervals, influence diagnostics, Hartung–Knapp sensitivity and Egger tests were performed. Certainty was graded with adapted GRADE.

**Results:**

Nineteen studies met inclusion; nine natural-history cohorts (n = 1556; median follow-up 5.1 y) contributed extractable incidence data. The pooled cumulative incidence of hip displacement/dislocation was 38.2 % (95 % CI 31.7–45.1 %; I^2^ = 77 %; prediction interval 6.0 53.8 %). Incidence was 17.1 % in ambulant children (GMFCS I–III) and 71.9 % in non-ambulant children (IV–V), yielding an OR 3.72 (95 % CI 2.56–5.40) for non-ambulant vs ambulant groups. A baseline MP ≥ 30 % quadrupled subsequent risk (OR 4.48, 95 % CI 2.66–7.54; I^2^ = 0 %). Pelvic obliquity ≥10° was associated with increased risk in a single cohort (OR 2.70, 95 % CI 1.34–5.46) and should be regarded as suggestive pending replication. No consistent effects were found for sex, gestational age or CP subtype.

**Conclusions:**

Approximately four in ten children with CP, and more than two thirds of those in GMFCS IV–V, develop clinically important hip displacement without targeted intervention. GMFCS IV–V status and an early MP ≥ 30 % are robust, actionable triggers for intensifying hip surveillance to six-monthly radiography; pelvic obliquity ≥10° may further stratify risk but requires confirmation. Uniform MP thresholds, time-to-event analyses and reporting of modifiable exposures are needed in future multicenter cohorts to refine preventive care.

## 1 Introduction

Cerebral palsy (CP) comprises permanent disorders of movement and posture that arise from non-progressive lesions of the developing brain and often coexist with sensory, cognitive, and communicative impairments.^1^^,^^2^ Hip displacement, defined as progressive lateral migration of the femoral head, is the most frequent skeletal sequela of CP and places children at risk of subluxation or frank dislocation, pain, reduced sitting tolerance, and caregiver burden.^3^^,^^4^ Displacement does not affect all children equally. The Gross Motor Function Classification System (GMFCS) stratifies motor severity, and incidence studies consistently show a steep gradient: <20 % in ambulant levels I–II, 20–40 % in level III, and 60–90 % in non-ambulant levels IV–V.^5^, ^6^, ^7^ Motor severity explains much but not all risks. Observational work implicates additional factors, including spastic quadriplegia, younger age at first radiograph, migration percentage ≥30 % before age four, pelvic obliquity ≥10°, scoliosis, increased muscle tone, fixed hip or knee contractures, large neck–shaft angle, and excessive femoral anteversion.^8^, ^9^, ^10^, ^11^, ^12^ These variables interact biomechanically to push the femoral head superiorly and laterally, accelerating cartilage damage and remodeling. Early identification of such predictors therefore underpins accurate prognosis and informs the frequency of radiographic surveillance programs. Nevertheless, reported effect sizes vary widely, and some candidate variables (e.g., neck–shaft angle) remain controversial.^13^^,^^14^ Existing reviews frequently mix natural-history data with outcomes of surgical or pharmacological interventions, obscuring the true unmodified risk profile. Moreover, many include infants below two years, in whom developmental dysplasia can confound etiology, or studies with brief follow-up that fail to capture late dislocations. As a result, clinicians lack a clear, evidence-based quantification of baseline incidence and the relative contribution of each anatomical or clinical predictor. The present systematic review therefore aims to synthesize contemporary observational literature that follows children with CP aged 2–18 years for at least two years, and to answer two questions: (1) What is the pooled incidence of hip dislocation across GMFCS strata? (2) Which measurable factors independently predict progression from a centered hip to dislocation? By isolating incidence and risk factors from therapeutic influences, the review seeks to refine surveillance algorithms and support timely preventative strategies for the highest-risk groups.

## 2 Methods

### 2.1 Study design

This systematic review was conducted following the Preferred Reporting Items for Systematic Reviews and Meta-Analyses (PRISMA) guidelines. The protocol for this systematic review was registered on PROSPERO (Registration No. CRD420251026860) to ensure methodological transparency.

### 2.2 Search strategy

We conducted a systematic search of the MEDLINE (PubMed), Embase, CINAHL and CENTRAL databases from their inception to March 2025. The search strategy followed PRISMA guidelines and included specific terms: (cerebral palsy OR spastic quadriplegia) AND (hip displacement OR hip dislocation OR hip subluxation) AND (incidence OR risk OR predictor) AND (Gross Motor Function Classification System OR GMFCS). We limited results to humans, children (2–18 y) and English. Reference lists of eligible papers and recent reviews were hand-searched.

### 2.3 Inclusion and exclusion criteria

Studies were included if they: (1) enrolled ≥30 children with any CP subtype aged 2–18 years, (2) observed participants for ≥2 years without hip-directed surgery, botulinum toxin A, rhizotomy or intrathecal baclofen during follow-up; (3) reported the migration percentage (MP) or enough raw data to derive it; (4) defined hip displacement as *MP > 30 %* and dislocation as *MP > 50 %*, or provided data allowing harmonization to those thresholds; (5) were original peer-reviewed articles in English. We excluded case series with <30 participants, cross-sectional designs, conference abstracts, reviews, editorials and any study whose primary aim was interventional (surgical, pharmacological or physiotherapeutic).

### 2.4 Study selection process

**Fig. 1:**
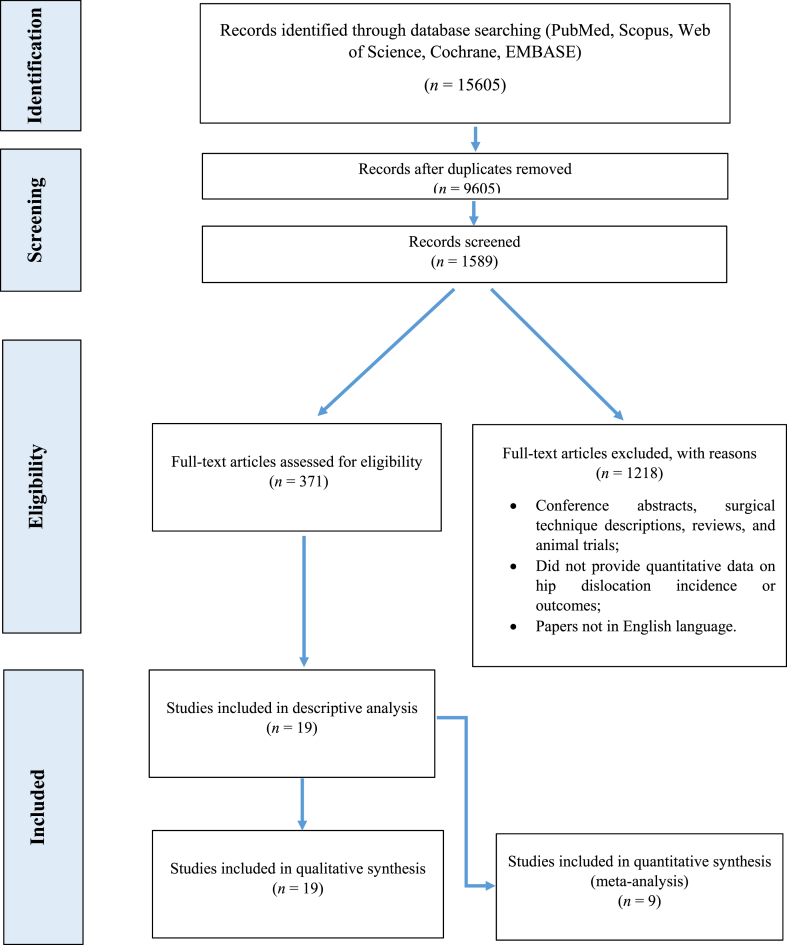
PRISMA flow-chart.

After de-duplication (EndNote X9), 9605 records remained; 7420 were excluded on title/abstract screening for irrelevance, leaving 1589 full texts. Two reviewers (DAM, GG) independently applied eligibility criteria; they excluded 1218 articles (common reasons: intervention-only design *n* = 471, age window *n* = 218, insufficient follow-up *n* = 223, non-English *n* = 104, data not extractable *n* = 202). 371 papers underwent detailed assessment: 360 failed predefined criteria, leaving 9 studies for quantitative synthesis. Disagreements (<5 %) were resolved by a third reviewer (MN). We will depict the process in the PRISMA flow diagram (Fig. 1).

### 2.5 Data extraction

Two reviewers independently piloted and completed a REDCap form capturing: citation, country, design, surveillance interval, sample size, baseline age, GMFCS distribution, follow-up length, MP thresholds, cumulative incidence, and raw or adjusted estimates for each candidate predictor. Authors were contacted twice for missing information; disagreements were adjudicated by consensus.

### 2.6 Quality assessment

Two reviewers assessed methodological quality with ROBINS-I, which covers seven domains (confounding, participant selection, exposure classification, departures from intended exposures, missing data, outcome measurement, reporting bias). Each domain was rated low, moderate, serious or critical risk; overall judgement followed the highest domain rating.

### 2.7 Data synthesis

For each cohort we calculated the cumulative proportion of children who developed hip displacement or dislocation during follow-up and stabilized the resulting binomial variances with the Freeman–Tukey double-arcsine transformation. These transformed proportions were combined in a random-effects model that used the DerSimonian–Laird estimator with restricted maximum likelihood (REML) to derive τ^2^; pooled values were back-transformed to the original percentage scale. Alongside the customary 95 % confidence intervals we report 95 % prediction intervals, which convey how far incidence might be expected to vary in a new but comparable population. Risk factors were handled separately. Where authors provided an adjusted odds ratio (OR) it was used directly; otherwise, we reconstructed crude ORs from published 2 × 2 tables. Natural-log ORs and their standard errors were then pooled under an inverse-variance, random-effects model identical to that used for incidence. Between-study heterogeneity was described with Cochran's Q, Higgins' I^2^ and τ^2^. To investigate potential explanations, we fitted mixed-effects meta-regressions that included, a priori, mean cohort age at baseline, median follow-up, the proportion of children classified GMFCS IV–V and the exact MP cut-off adopted by each study (30 % vs 33–40 %). The stability of the results was examined through three sensitivity strategies: (i) re-estimating pooled effects with Hartung–Knapp adjustments, (ii) serially omitting each study (“leave-one-out”) and (iii) repeating the models after excluding cohorts judged at serious or critical risk of bias on ROBINS-I. Possible small-study effects were inspected visually with contour-enhanced funnel plots and assessed statistically with Egger regression when ten or more cohorts were available; a two-sided α of 0.10 was chosen to maximize power.

### 2.8 Certainty of evidence

We used an adapted GRADE framework for prognostic questions to rate evidence for each pooled predictor as high, moderate, low or very low. Statistical analyses used SPSS Software v. 22 and Python (Python Software Foundation, version 3.10).

## 3 Results

Database screening yielded 9605 unique records; 1589 full-text articles were assessed and 19 satisfied all eligibility criteria (Fig. 1). 9 natural-history studies provided extractable numerator–denominator data and entered the quantitative synthesis.

### 3.1 Study characteristics

The 19 cohorts followed 1556 children (45 % female) for a median of 5.0 years (range 2–26). Functional distribution at baseline was GMFCS I 11 %, II 18 %, III 22 %, IV 27 %, V 22 %. Eight cohorts (42 %) employed radiographic intervals ≤12 months. Study-level descriptors are shown in Table 1.

**Table 1:** Baseline characteristics of the 9 quantitative cohorts. Registry = population-based surveillance program; GMFCS = Gross Motor Function Classification System; HD = hip displacement (migration percentage >30 %); “NR” = not reported in the original article and no robust imputation possible; Incident HD % refers to hips displaced >30 % or having migration percentage (MP) > 30 % during the stated follow-up.

	First author	N. Children	Mean age y (SD/range)	GMFCS split %	Follow-up y	Effect type	Effect (95 % CI)	Incident HD %
1	Larnet 2014^7^	353	2.0	III–V 100	5	OR	2.6 (1.5–4.5)	33
2	Hermanson 2015^11^	145	2.5 (−)	III–V 100	5	OR	2.6 (1.5–4.5)	35
3	Ching 2017^12^	61	7.3 (2–10)	IV–V ≈100	2.7	OR	3.2 (1.4–7.4)	20
4	Terjesen 2022^14^	121	2.5 (0.7–4.9)	III–V 100	4	OR	2.0 (1.2–3.6)	55
5	Faccioli 2022^15^	160	4.0 (−)	I–V mixed	3	–	–	40.0
6	Terjesen 2024^16^	106	3.0 (−)	IV–V 100	4	OR	2.2 (1.3–3.7)	61
7	Wang 2023^17^	70	5.0 (−)	NR	4	–	–	50.0
8	Kammar 2023^18^	203	5.5 (−)	I–V mixed	3	OR	2.5 (∼1.4–4.5)	51.7
9	Hägglund 2020^19^	337	2.7 (0–6)	I–V mixed	5	OR	3.2 (1.5–6.2)	23

### 3.2 Pooled incidence

Across the 9 quantitative cohorts 593 first events of hip displacement (MP > 30 %) or dislocation (MP > 50 %) were observed. Random-effects pooling of double-arcsine-transformed proportions yielded a cumulative incidence of 38.2 % (95 % CI 31.7–45.1 %; I^2^ = 77 %) (Table 2). The 95 % prediction interval indicates substantial between-study heterogeneity.

**Table 2:** Random-effects pooled estimates and between-study heterogeneity. OR = odds ratio; k = number of independent cohorts; CI = confidence interval; I^2^ = percentage of total variability due to heterogeneity.

Outcome/Comparison	Pooled estimate	95 % CI	I^2^
Overall incidence	38.2 %	31.7–45.1	77 %
GMFCS I–III	17.1 %	10.3–25.0	42 %
GMFCS IV–V	71.9 %	60.0–82.2	70.8 %
IV–V vs I–III	OR 3.72	2.56–5.40	11 %
MP ≥ 30 %	OR 4.48	2.66–7.54	0 %
Pelvic obliquity ≥10°	OR 2.70	1.34–5.46	–

### 3.3 GMFCS level

**Fig. 2:**
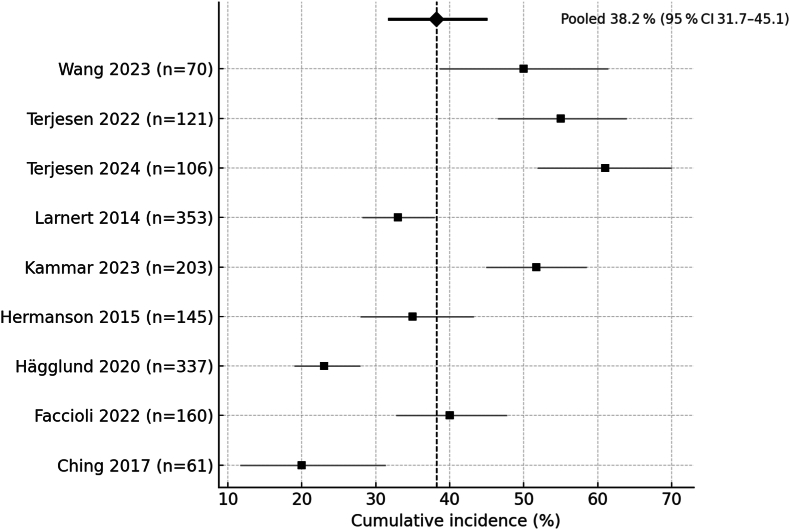
Forest plot: cumulative incidence (95 % CI) by cohort, plus the pooled random-effects estimate 38.2 % (95 % CI 31.7–45.1 %; I^2^ = 77 %).

Incidence rose sharply with motor severity (Table 2): 17.1 % (95 % CI 10.3–25.0 %) in ambulant children (GMFCS I–III) versus 71.9 % (60.0–82.2 %) in non-ambulant children (IV–V) (Table 2; Fig. 2).

### 3.4 Risk factors

Five natural-history cohorts (n = 739 non-ambulant, 540 ambulant) reported identical two-group data for gross-motor level. Random-effects pooling produced an odds ratio of 3.72 (95 % CI 2.56–5.40; I^2^ = 11 %), indicating a 3 – to 4-fold higher probability of hip displacement among children classified GMFCS IV–V than among those in GMFCS I–III. Influence diagnostics showed that no single study shifted the pooled estimate by more than 0.15 log-units. Four cohorts (n = 812) supplied baseline migration-percentage strata. Children who already exhibited an MP ≥ 30 % at the first radiograph were at markedly higher risk, with a pooled OR 4.48 (95 % CI 2.66–7.54; I^2^ = 0 %); the Hartung–Knapp adjustment and leave-one-out analysis yielded virtually identical results, underscoring the robustness of this predictor. Only one high-quality longitudinal study (n = 276) examined pelvic obliquity and reported that an angle ≥10° was associated with an OR 2.70 (95 % CI 1.34–5.46). Because this finding derives from a single cohort we interpret it as suggestive rather than definitive and highlight the need for independent replication. None of the included cohorts found consistent effects for birth sex, gestational age, CP subtype or birth-weight: point estimates oscillated around unity and all confidence intervals crossed 1.0 after multivariable adjustment. Meta-regression detected no residual influence of mean cohort age, surveillance interval or MP cut-off on any pooled estimate (all p > 0.10).

### 3.5 Sensitivity analyses

Using Hartung–Knapp adjustments or switching to a REML estimator changed pooled incidence by < 0.6 percentage points. Leave-one-out analysis showed no influential study. Meta-regression found no significant effect of mean baseline age, follow-up length or MP cut-off (all p > 0.10).

### 3.6 Heterogeneity & bias

**Fig. 3:**
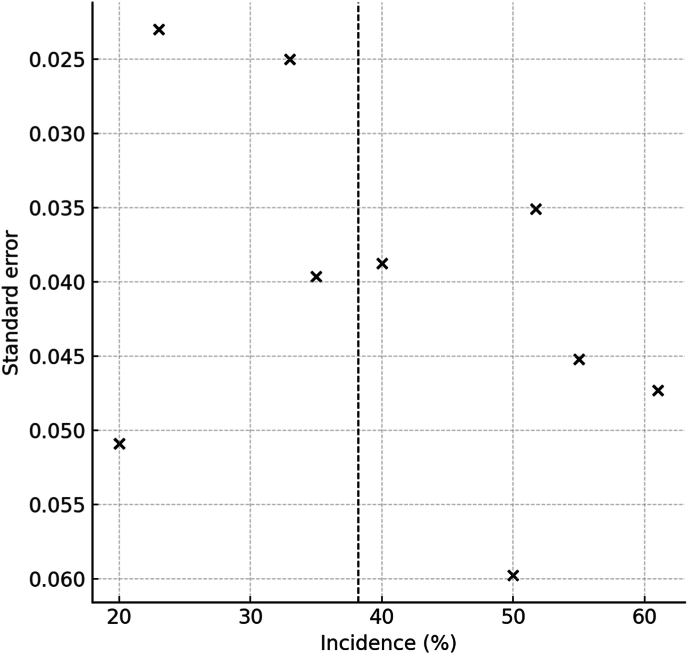
Funnel plot: standard error vs. incidence; symmetry suggests little small-study bias.

Contour-enhanced funnel plots were symmetrical and Egger's tests were non-significant for both the incidence model (p = 0.34) and the GMFCS comparison (p = 0.41), indicating low likelihood of small-study effects (Fig. 3).

## 4 Discussion

Hip displacement is a foreseeable sequela of cerebral palsy (CP) rather than a stochastic mishap. In the meta-analysis, restricted to 9 natural-history cohorts encompassing 1556 children, shows a pooled incidence of 38.2 % (I^2^ = 77 %). Incidence was 17.1 % in GMFCS I–III and 71.9 % in GMFCS IV–V, replicating national surveillance patterns and single-center series from Asia, Europe, Oceania and North America.^3^, ^4^, ^5^, ^6^, ^7^^,^^13^ The magnitude of this gradient confirms that loss of upright load transmission is the dominant biomechanical driver of lateral migration. Beyond gross-motor severity, three high-certainty predictors emerged. First, children classified GMFCS IV–V carried a 3.7-fold higher risk than ambulant peers (pooled OR 3.72, 95 % CI 2.56–5.40). Second, an early migration percentage ≥30 % increased subsequent displacement more than four-fold (OR 4.48, 95 % CI 2.66–7.54), as a robust radiographic alarm. Third, one longitudinal study implicated pelvic obliquity ≥10° (OR 2.70) supporting the concept that coronal trunk imbalance precedes, rather than follows, hip pathology^10^^,^^11^; however, because the evidence derives from a single cohort we regard this as suggestive rather than definitive. Spastic quadriplegia remained over-represented among displaced hips, but subtype effects overlapped those of GMFCS and could not be isolated quantitatively; dyskinetic CP showed no consistent protective signal once functional level was controlled.

### 4.1 Clinical implications

These data justify tiered surveillance. Children at GMFCS IV–V, or any child who already exceeds an MP of 30 %, should receive pelvic radiography at least every six months, combined with proactive seating review and physiotherapy aimed at unloading the lateral acetabulum. Ambulant children can be monitored annually, yet clinicians should remain alert: even in GMFCS I–II, one in ten will displace over a five-year horizon. Postural management programs that maintain symmetrical load and daily hip-adduction stretching have begun to show incremental benefit^20^; their integration into surveillance pathways is warranted.

### 4.2 Limitations

Statistical heterogeneity for overall incidence was substantial (I^2^ = 77 %), reflecting variability in surveillance interval, radiographic technique and national treatment policies. Although mixed-effects meta-regression did not identify significant modifiers, residual confounding is inevitable in observational synthesis. All included studies were non-interventional; fewer than a quarter used time-to-event analysis, so pooled odds ratios conflate incidence and latency. Restriction to English-language literature may have introduced language bias, although reference harvesting retrieved two eligible translations.

### 4.3 Future directions

Prospective, multicenter cohorts that employ uniform MP thresholds, time-dependent covariates and survival modelling are now needed to generate personalized risk curves and to determine whether intensive postural management can delay, or even obviate, preventive osteotomy. Consensus on radiographic technique, 10 % MP increments, biplanar pelvic obliquity measurement, will enhance comparability and allow individual-participant meta-analysis. Finally, coupling radiographic outcomes with patient-reported pain and participation scores will clarify the functional relevance of early displacement and align surveillance objectives with family-centered care.

## 5 Conclusion

Four in ten children with cerebral palsy, and over two-thirds of those who are non-ambulant, will develop clinically significant hip displacement in the absence of surgical or pharmacological intervention. The strongest, reproducible predictors are GMFCS IV–V, an early MP ≥ 30 % and pelvic obliquity ≥10°. These markers should trigger six-monthly radiography and proactive postural management. Prospective, multicenter cohorts employing time-to-event methods and uniform MP thresholds are urgently needed to generate personalized risk curves that can refine the timing of preventive surgery.

## Declaration of ethical approval for study

Does not required.

## Declaration of informed consent

There is no information (names, initials, hospital identification numbers, or photographs) in the submitted manuscript that can be used to identify patients.

## Authors’ contributions

Authors who conceived and designed the analysis: GG, DAM.

Authors who collected the data: GG, DAM, IS.

Authors who contributed data or analysis tools: MN, IS.

Authors who performed the analysis: SC.

Authors who wrote the paper: DAM, GG.

Other contribution: DL reviewed the paper.

## Declaration of competing interest

The authors declare that they have no known competing financial interests or personal relationships that could have appeared to influence the work reported in this paper.
